# Transcriptomics and Proteomics Identify Serotonin Transporter as a Promising Therapeutic Target for Essential Tremor

**DOI:** 10.1016/j.mcpro.2025.101442

**Published:** 2025-11-04

**Authors:** Lingbing Wang, Zhuofan Zhou, Suzhen Lin, Yanjing Li, Shaoyi Zhang, Tian-Le Xu, Xing-Lei Song, Yiwen Wu

**Affiliations:** 1 Department of Neurology and Institute of Neurology, Ruijin Hospital, Shanghai Jiao Tong University School of Medicine, Shanghai, China; 2 Department of Anatomy and Physiology, Shanghai Jiao Tong University School of Medicine, Shanghai, China; 3 Department of Anesthesiology, Songjiang Research Institute, Shanghai Key Laboratory of Emotions and Affective Disorders, Songjiang Hospital Affiliated to Shanghai Jiao Tong University School of Medicine, Shanghai, China

**Keywords:** essential tremor, transcriptomic sequencing, proteomic analysis, serotonin transporter, therapeutic target

## Abstract

Essential tremor (ET) stands as one of the most prevalent movement disorders originating from cerebellar dysfunction. However, effective treatment remains limited, largely due to a poor understanding of its molecular pathology. The harmaline-induced tremor in mice is a well-established model for ET research, though its mechanisms remain unclear. This study aimed to get insight into the molecular intricacies underlying cerebellar dysfunction in this model. Combining LC-MS/MS and RNA-Seq approach, we delved into the cerebellar alterations in harmaline-induced tremor in mouse. Multi-omics profiling identified 5194 correlated coding molecules, among which 19 were significantly dysregulated. Further KEGG enrichment analysis identified cerebellar serotonin transporter (SERT) as the key molecule in harmaline-induced tremor. We validated the upregulation of SERT in the cerebellar cortex following harmaline induction, particularly within Purkinje cells, and demonstrated that pharmacological inhibition or genetical knockdown of SERT significantly attenuated tremor severity and neuronal hyperexcitability. Further mechanistic studies revealed that harmaline-induced SERT upregulation leads to depleted serotonin levels in the cerebellum, contributing to tremor pathogenesis. In general, our study unveils crucial insights that could pave the way for molecular target identification and effective therapeutic interventions for ET.

## Highlights

• Transcriptomic and proteomic analyses detected the up-regulation of SERT in cerebellar cortex in harmaline-induced essential tremor.
• Multiple experiments confirmed the pivotal role of SERT in harmaline-induced essential tremor.
• Drug-induced SERT depletion and its genetical knockdown suggests its potential role in the novel therapeutical methods for essential tremor.

## Graphical Abstract

**Figure:**
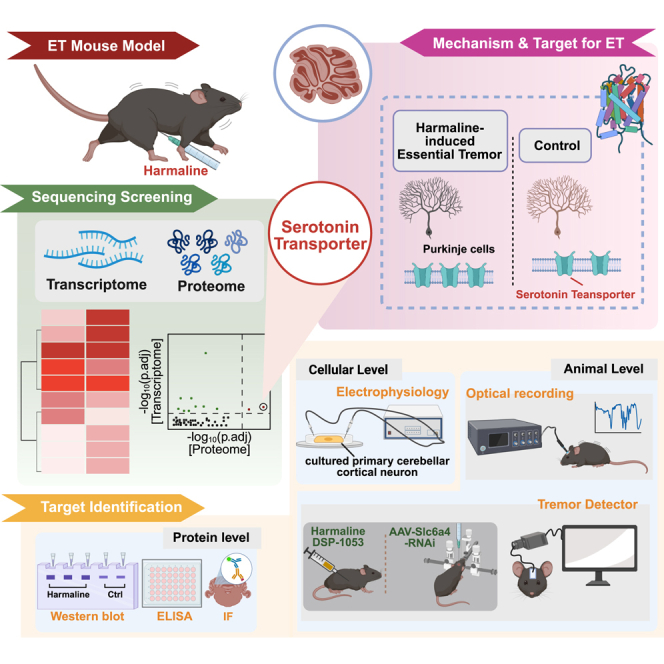

## In Brief

The detailed mechanism of essential tremor remains unclear. This study combined transcriptomic and proteomic profiling of the cerebellum in a harmaline-induced essential tremor model. They identify serotonin transporter (SERT) as a key upregulated protein. SERT upregulation depletes cerebellar serotonin, increasing Purkinje cell excitability and driving tremor. Crucially, both pharmacological and genetic inhibition of SERT attenuates tremor severity, revealing SERT as a promising therapeutic target for essential tremor.

Essential tremor (ET) is one of the most common movement disorders (1, 2), characterized by the rhythmic oscillation of agonist and antagonist muscle groups, typically occurring at a frequency of 8 to 12 Hz (3, 4). The incidence of ET has been reported to be around 0.9%, with a significant increase in prevalence among individuals over 65 years old, reaching 4.6% (5). Drugs for ET treatment include propranolol, a β-adrenergic receptor antagonist, and primidone, an anticonvulsant. However, these medications are associated with severe side effects and provide limited therapeutic benefit, resulting in an average tremor reduction of only around 50%. Consequently, there is a pressing need for targeted therapies that explore new biological pathways. Nevertheless, the underlying mechanisms of ET remain elusive. The harmaline-induced tremor is a recognized model used for screening potential therapies for ET (6). In laboratory animals, a tremor at a frequency of 8 to 12 Hz can be generated after intraperitoneal injection of harmaline at doses ranging from 10 to 50 mg/kg, with a time latency of 3 to 10 min (7). Furthermore, Louis *et al.*, discovered elevated concentrations of harmane, another compound of harmala alkaloids, in patients with ET compared to controls in the Faroe Islands (8). These findings suggest that β-carboline alkaloids may contribute to ET pathogenesis in both humans and laboratory animals. However, the exact pathological mechanisms underlying harmaline-induced tremor have not been conclusively elucidated (9).

The prevailing viewpoint proposes that harmaline can elicit abnormal activation of climbing fibers (CF) within the inferior olivary nucleus (ION) of the cerebellum, leading to the formation of aberrant synapses with Purkinje cells (PC) in the cerebellar cortex (10). Recent findings indicate that harmaline induces a burst-firing pattern in PC and that the absence of PC neurotransmission can attenuate harmaline-induced tremor (10). These results underscore the crucial role of the cerebellar cortex itself, particularly the Purkinje cells, in harmaline-induced tremor. Since the harmaline-induced tremor mouse model serves as a pharmacological assessment of potential ET treatments, understanding the mechanisms through which harmaline triggers tremor in the cerebellum may facilitate the development of novel therapeutic strategies for ET.

Transcriptomic analysis has been widely recognized as an efficient method for unveiling tissue-specific alterations, including gene splicing, structural variations and transcriptional changes (11). This approach is capable of uncovering variations in gene expression (12). However, the significance of post-transcriptional protein turnover in determining protein function should not be underestimated (13). The unique advantage of combined analyses of transcriptomics and proteomics lies in their complementary integration, which helps to mitigate systematic biases inherent in each method independently.

In this study, we employed a comprehensive approach integrating both transcriptomic and proteomic analysis to thoroughly disclose the alteration in the cerebellum induced by harmaline treatment. To gain deeper insight into the cerebellar mechanisms contributing to harmaline-induced tremor, we conducted a comparative analysis of the cerebellums of mice treated with harmaline and their control counterpart. Through the integration of transcriptomic results and proteomic profiles, we identified numerous candidate genes. Subsequent extended biological experiments were conducted to validate the role of a candidate molecule serotonin transporter (SERT), in the activity of cerebellar PC during harmaline-induced tremor. Thus, this study reveals a novel molecular mechanism for harmaline-induced ET and identifies a potential therapeutic target.

## Experimental procedures

### Experimental Design and Statistical Rationale

This study initially compared 3 harmaline-injected mice with 3 control mice (injected with DMSO diluted with saline) for distinct behavioral phenotypes. Subsequently, the cerebellums from the harmaline-induced tremor mice and control mice were split; one-half of each cerebellum was used for transcriptomic analysis and the other for proteomic analysis. Significantly changed proteins identified by LC-MS/MS that were also identified as significant in the transcriptomic results were considered the integrated dataset for further verification.

Verification experiments were based on the combined data and included:

Western blot: Analysis of 3 harmaline-injected and 3 control cerebellar cortex samples, with each experiment performed in triplicate and averaged for final statistical comparison.

5-HT detection: Measurement of serotonin dynamics in the cerebellar cortex of 4 mice injected with AAV-hSyn-5HT3.0.

Cell electrophysiology: Recordings from 5 cultured cerebellar cortex neurons in each group (control, harmaline and harmaline + DSP-1053), Immunofluorescence: Comparison of SERT in Purkinje cells comparison between control (n = 15) and Harmaline-treated (n = 10) mice.

Behavioral experiments on pharmacological inhibition of SERT: 8 mice for the control group, 3 mice for harmaline group, and 3 for harmaline + DSP-1053 group.

Behavioral experiments on genetic manipulation of SERT: in mice with AAV2-Slc6a4-RNAi injection for SERT knockdown, 7 mice for the control group and 9 mice for harmaline group.

### Tremor Detection in Freely Moving Mice

Tremors were recorded in freely moving mice using a trer detector system (Medusa, bio-signal), by pre-implanting an electrode slice on the skull of the mice. During tremor measurement, the electrode was connected to a signal transformer, enabling the recording of behavioral activity and transforming vibratory signals into digital signals for subsequent analysis. The collected data were analyzed using a preset program based on the power spectrum density function, followed by transformation into the frequency domains. Spectrum data were normalized through logarithmic (lg) calculation. Adult mice were administered 30 mg/kg harmaline (Topscience, T2792) via intraperitoneal injection (i.p.). For specific pharmacological interventions, mice were administered DSP-1053 (10 mg/kg), an antagonist for SERT, or an equal amount of saline as a control. DSP-1053 was delivered to mice (i.p.) 1 h prior to harmaline administration, ensuring that blood concentration of the compound approached its peak at the time of harmaline exposure (14). The mice were then sacrificed 30 min after harmaline injection.

### Animals

All animal care and experimental procedures were approved by the Animal Ethics Committee of Shanghai Jiao Tong University School of Medicine and by the Institutional Animal Care and Use Committee (Department of Laboratory Animal Science, Shanghai Jiao Tong University School of Medicine; policy DLAS-MP-ANIM.01–05). All mice were group-housed under controlled conditions of lighting (12-h light/12-h dark cycle) and temperature (22 ± 2 °C), with rodent chow and water ad libitum. Adult male C57BL/6 mice (8-week-old) were used for all experiments.

Analyses were conducted on 8-week-old adult male C57BL/6 mice that received intraperitoneal injections of harmaline and DSP-1053, along with their wild-type littermates serving as controls (injected with saline). 30 min after injection, the animals were anesthetized with isoflurane and subsequently euthanized, followed by decapitation for brain extraction. Cerebellar cortex samples were promptly dissected and either flash-frozen in liquid nitrogen for LC-MS/MS or preserved in RNALater (Beyotime, R0118) for subsequent transcriptomic sequencing.

### Protein Extraction and Digestion

Sample lysis and protein extraction were conducted using SDT buffer (4%SDS, 100 mM Tris-HCl, 1 mM DTT, pH7.6). The amount of protein was quantified with the BCA Protein Assay Kit (Bio-Rad). Trypsin was used for protein digestion following filter-aided sample preparation (FASP) procedure described by Mann *et al*. (15) (detailed procedure is described as follows). The digest peptides of each sample were desalted on C18 Cartridges (Empore SPE Cartridges C18 (standard density), bed I.D. 7 mm, volume 3 ml, Sigma), then concentrated by vacuum centrifugation and reconstituted in 40 μl of 0.1% (v/v) formic acid.

### Filter-Aided Sample Preparation (FASP Digestion) Procedure

30 μl SDT buffer (4% SDS, 100 mM DTT, 150 mM Tris-HCl pH 8.0) was added into 200 μg of proteins of each sample. UA buffer (8 M Urea, 150 mM Tris-HCl pH 8.0) along with repeated ultrafiltration (Microcon units, 10 kD) was applied to remove the impurity, including the detergent, DTT, and other low-molecular-weight components. After addition of 100 μl iodoacetamide (100 mM IAA in UA buffer) for blocking reduced cysteine residues, the samples were then incubated for 30 min (in the dark). Before the final digestion by trypsin (Promega, 4 μg, in 40 μl 25 mM NH4HCO3 buffer overnight at 37 °C), the filters were washed with 100 μl UA buffer three times and then 100 μl 25 mM NH4HCO3 buffer twice. The digested peptides were collected as a filtrate and then desalted on C18 Cartridges (Empore SPE Cartridges C18 (standard density), bed I.D. 7 mm, volume 3 ml, Sigma), concentrated by vacuum centrifugation and reconstituted in 40 μl of 0.1% (v/v) formic acid. 280 nm UV light spectral density was then used to estimate the peptide content, with an extinction coefficient of 1.1 of 0.1% (g/l) solution. 100 μg peptide mixture of each sample was labeled with TMT reagent (Thermo Scientific).

### High pH Reversed-Phase Fractionation

High pH Reversed-Phase Peptide Fractionation Kit (Thermo Scientific) was applied to fractionate the peptides with TMT labelling. The dried peptide mixture was reconstituted and acidified with TFA solution (0.1%) and loaded to the equilibrated, high-pH, reversed-phase fractionation spin column. Peptides were bound to the hydrophobic resin and desalted in column under low-speed centrifugation. Finally, the peptides were separated into 10 different fractions by a step gradient of increasing acetonitrile concentrations in a volatile high-pH elution solution. The collected fractions were then got desalted on C18 Cartridges and concentrated by vacuum centrifugation.

### LC-MS/MS Analysis

Q Exactive mass spectrometer (Thermo Scientific) coupled with Easy nLC (Proxeon Biosystems, now Thermo Fisher Scientific) for 60/90 min was used for LC-MS/MS analysis. The peptides were loaded onto a reverse phase trap column (Thermo Scientific Acclaim PepMap100, 100 μm∗2 cm, nanoViper C18) connected to the C18-reversed phase analytical column (Thermo Scientific Easy Column, 10 cm long, 75 μm inner diameter, 3 μm resin). In this system, buffer A (0.1% Formic acid) was used for the column balance, and a linear gradient of buffer B (84% acetonitrile and 0.1% Formic acid, flow rate: 300 nl/min, controlled by IntelliFlow technology) was used for separation. The mass spectrometer was operated in positive ion mode. The most abundant precursor ions were automatically chosen by the survey scan (300–1800 m/z) for HCD fragmentation. Automatic gain control (AGC) target was set to 3e6, and maximum inject time to 10 ms, dynamic exclusion duration to 40.0 s. Survey scans were acquired at a resolution of 70,000 at 200 m/z and resolution for HCD spectra was set to 17,500 at 200 m/z, and isolation width was 2 m/z. Normalized collision energy was 30 eV and the underfill ratio was defined as 0.1%.

### Identification and Quantitation of Proteins

MASCOT engine (Matrix Science; version 2.2) and Proteome Discoverer 1.4 software were applied for searching the MS raw data and further quantitation analysis. Related parameters and instructions are as follows: max missed cleavages: 2, peptide mass tolerance was set at ± 20 ppm, fragment Mass Tolerance at 0.1 Da, Fixed modifications at Carbamidomethyl (C), TMT 6/10/16 plex (N-term), TMT 6/10/16 plex (K), Variable modifications set as Oxidation (M), the database used for the searching was Swissprot_Mus_musculus_17063_20210106.fasta, database pattern as Decoy, the peptide FDR was set to be ≤ 0.01. The filtering criteria was set as Fold change>1.2 and *p* value<0.05. The protein ratios are calculated as the median of only unique peptides of the protein and normalized by median protein ratio (after the normalization, the median protein ratio should be 1).

### RNA Extraction and Transcriptomic Analysis

Paired-end libraries were prepared using an ABclonal mRNA-seq Lib Prep Kit (ABclonal). Total RNA was extracted from cerebellum cortex samples preserved in RNALater using TRIzol reagent. The RNA concentration was quantified at 260 nm using the RNA 6000 Nanodrop on the Agilent 4150 Bioanalyzer (Agilent). Afterward, mRNAs were isolated using beads with Oligo (dT) and subjected to random fragmentation buffer. First-strand cDNAs were synthesized with random hexamer primers and reverse transcriptase (RNase H) with mRNA fragments as templates, followed by second-strand cDNA synthesis using cDNA polymerase I, RNAseH, buffer and dNTPs. The synthesized double-stranded cDNA fragments were then adapter-ligated for preparation of the paired-end library. Complementary DNAs (cDNAs) were then used for PCR amplification and PCR products were purified by AMPure XP beads. The library quality was assessed on an Agilent Bioanalyzer 4150 system.

The sequencing was performed with an Illumina Novaseq 6000/MGISEQ-T7 instrument. And the data generated were then used for bioinformatics analysis (Shanghai Applied Protein Technology). Raw data generated was further processed and controlled by removing the adapter sequence and filtering out low quality (the number of lines with a string quality value less than or equal to 25 accounts for more than 60% of the entire reading) and N (the base information cannot be determined) ratio is greater than 5% reads to obtain the clean reads for subsequent analysis.

The clean reads were separately aligned to reference genome with orientation mode using HISAT2 software (http://daehwankimlab.github.io/hisat2/) to obtain mapped reads, during which, mouse GRCm39 was used for the alignment. And FeatureCounts (http://subread.sourceforge.net/) was used to count the reads numbers mapped to each gene, and the FPKM of each gene was calculated based on the length of the gene and reads count mapped to this gene. The differential expression analysis was performed using the DESeq2 (http://biocinductor.org/packages/release/bioc/html/DESeq2.html), DEGs with |log2fc| >1 and Padj <0.05 were considered to be significantly different expressed genes.

### The Combination Between Proteomic and Transcriptomic Results

Further, we combined the proteomic and transcriptomic sequencing results to systematically explore the outcomes of the harmaline treatment, first, we combined the transcriptomic and proteomic data that were from the same sample, when the proteins were identified in transcriptomic results, then shall be considered as correlated ones, the combined data was collected for the further analyzation.

### Bioinformatic Analysis

For cluster analyzation, hierarchical clustering analysis was performed by Cluster 3.0 (http://bonsai.hgc.jp/∼mdehoon/software/cluster/software.htm) and Java Treeview software (http://jtreeview.sourceforge.net). For subcellular localization, CELLO (http://cello.life.nctu.edu.tw/) was used to predict protein subcellular localization, for GO annotation, we used NCBI BLAST + client software (ncbi-blast-2.2.28+-win32.exe) to search the protein sequences, InterProScan to find homolog sequences, Blast2GO was then applied for mapping gene ontology (GO) terms and annotated sequencing. The GO annotation results were plotted by R scripts. For KEGG annotation, KAAS (KEGG Automatic Annotation Server) was used to mapped their pathway in KEGG. And for the enrichment analysis, based on the Fisher’ exact test, the whole quantified proteins were considered as background dataset. Benjamini- Hochberg correction for multiple testing was further applied to adjust derived *p*-values. And only functional categories and pathways with *p*-values under a threshold of 0.05 were considered as significant. All the identified proteins and the peptides information are listed in Supplementary 3.

### Western Blot

Fresh cerebellar cortex samples from mice were solubilized in lysis buffer containing proteinase inhibitors (Thermo Fisher, A32965) and phosphatase inhibitors (Thermo Fisher, 78420). After sonication and centrifugation, the supernatant was combined with loading buffer (Thermo Fisher, AM8547). Protein concentration was determined using the Pierce BCA Protein Assay Kit (Thermo Fisher, 23225). Following sample preparation, the proteins were loaded onto a 10% SDS-PAGE gel and transferred onto a PVDF membrane (Millipore). The membrane was blocked with 3% BSA (BioFroxx, 4240GR100), and then incubated with primary antibodies: SERT (1:1000, Abcam, ab102048) and GAPDH (Thermo Fisher, A300-639A-T). After an overnight incubation with the primary antibodies, the respective secondary antibodies (1:2000, Sigma-Aldrich, AP132P & AP124P) were applied. Signals were detected using the Tanon-5200 system (Tanon).

### ELISA Assay

An ELISA kit (MM-0443M1) was used to determine the concentration of serotonin in the cerebellum of harmaline-treated mice and their corresponding control counterpart. The cerebellum samples were washed, sonicated, and centrifuged with PBS (pH = 7.4, Sangon biotech, B548117-0500), and the resulting liquid supernatant was collected for further examination. The results were measured using a multi-mode microplate reader.

### Primary Culture of Cerebellar Cortical Neuron

At embryonic day 18 (E18), mice were anesthetized with diethyl ether. Following sterilization with 75% ethyl alcohol, a midline incision was made in the abdomen to expose and separate the uterus from the pregnant mice. Next, fetal mouse heads were extracted and placed in a Petri plate containing dissection buffer (DMEM + 5% penicillin/streptomycin). The scalps of the fetal mice were then cut open using sterilized scissors, and the brain tissues were carefully extracted. Under an optical microscope, the cerebellum was separated from the whole brain tissue, and the meninges were peeled off from the surface of the brain. The isolated cerebellum was placed in a 7 ml centrifuge tube with digestion buffer (2 ml 0.25% trypsin +2 ml dissection buffer). The centrifuge tube was transferred to a constant-temperature and humidity incubator for 15 min, with gentle shaking every 5 min during incubation. After incubation, excess digestion buffer was removed, and 4 ml of complete culture medium (DMEM + 10% FBS + 1% GlutaMAX) was added. The mixture was then homogenized with the digested brain tissue, filtered through a filter net, and the resulting cell suspension was centrifuged at 1000 rpm for 5 min. After centrifugation, the cell pellet was resuspended in 2 ml of complete culture medium. After cell counting, the cell suspension was added to cell-culture dishes preloaded with 2 ml of culture medium for cerebellar neurons (50% complete culture medium + 50% neural selective medium (Neuralbasal + 2% B27 + 1% GlutaMAX) + 2 μL T3 (20 mg/ml)). Cerebellar neurons were cultured in a constant-temperature and humidity incubator.

### Cell Electrophysiology

The electrophysiological characteristics of cultured cerebellar cortical neurons were recorded using voltage clamp techniques in the whole-cell mode of patch clamp at room temperature. Data acquisition and analysis were performed using the patch clamp amplifier system (MultiClamp 700B) and digital analog converter (Digidata 1550B and pClamp10). For the recording, glass electrodes filled with filtered electrode fluid (10 mM NaCl, 5 mM KCl, 1 mM MgCl_2_, 2 mM CaCl_2_, 10 mM HEPES, 10 mM Glucose, pH 7.4, 310–320 mOsm/L) were utilized. The resistance of the glass electrode, when immersed in the extracellular fluid (150 mM NaCl, 5 mM KCl, 1 mM MgCl_2_, 2 mM CaCl_2_, 210 mM glucose), ranged between 2 to 5 MΩ. Following baseline normalization, the glass electrode was connected to the cultured cerebellar cortical neuron under negative pressure. Then, the cell membrane was aspirated until it broke, achieving high-pressure sealing and establishing the whole-cell mode of patch clamp.

### Virus Injection and Optical Recording

The mouse was anesthetized with isoflurane. Then, the mouse’s head was restrained in a stereotaxic instrument, and the scalp above the cerebellar region was incised following fur removal. Adequate sterilization was applied, and a hole was drilled in the skull. A Hamilton syringe containing the virus (WZ Biosciences, AAV-hSyn-5HT3.0) was inserted into the mouse cerebellum with the coordinates (relative to Bregma: AP -6.75 mm, ML 1.8 mm, DV -2.5 mm). A total of 300 nl of the virus, administered at a rate of 0.1 μL per minute, was injected into the cortex of the mouse’s cerebellum. Ten minutes after completing the injection, the syringe was gradually removed at a rate of 0.05 mm per minute. Subsequently, an optogenetic fiber (Thinker Tech Nanjing Biotech Co., Ltd) was implanted above the virus-injection area (relative to Bregma: AP -6.75 mm, ML 1.8 mm, DV: −2 mm). Following these procedures, the scalp was sutured, and the mice received appropriate post-operative care. Upon full recovery and viral transgene expression, the optical signal of serotonin was detected using Signal-channel Fiber Photometry (Thinker Tech Nanjing Biotech Co., Ltd).

### Immunofluorescence

Mice were transcardially perfused with phosphate-buffered saline (PBS) followed by 4% paraformaldehyde (PFA) in PBS. Brains were then removed and post-fixed in 4% PFA for 24 h at 4 °C. Subsequently, tissues were embedded in 3% agarose and sectioned at 30 μm using a vibratome (Leica VT1000). Sections were stored in PBS at 4 °C until use.

For staining, free-floating sections were placed in 24-well plates and permeabilized with 0.1% Triton X-100 for 10 min. After blocking with 5% bovine serum albumin (BSA) for 1 h at room temperature (RT), sections were incubated overnight at 4 °C with primary antibodies diluted in 1% BSA. The following primary antibodies were used: anti-calbindin (1:500, Proteintech, Cat # 66394-1-Ig) and anti-SERT (1:500, Alomone Labs, AMT-004). After washing with PBS, sections were incubated with Alexa Fluor 488- or 568-conjugated secondary antibodies (1:500, Invitrogen, A-11001, A-11011) for 1 h at RT. Nuclei were counterstained with DAPI (1 μg/ml, Invitrogen, Cat # 62248) for 20 min. Finally, sections were mounted on slides with antifade mounting medium (SouthernBiotech, Cat # 0100-01) and imaged using a confocal microscope (Nikon). All images were acquired under identical settings for control and experimental groups.

### SERT Knockdown Virus Injection and Tremor Analysis

Mice were anesthetized with isoflurane and placed in a stereotaxic frame (RWD, China). After fur removal, the scalp above the cerebellar region was incised, and the surgical field was sterilized. Small holes were drilled in the skull above the target sites, and a Hamilton syringe loaded with the viral solution was lowered into the cerebellum at the following coordinates relative to Bregma:

AP −6.75 mm, ML +1.8 mm, DV −2.5 mm

AP −6.75 mm, ML −1.8 mm, DV −2.5 mm

AP −6.75 mm, ML 0 mm, DV −2.5 mm.

At each site, 200 nl of viral solution (AAV2-Slc6a4-RNAi (106098-1), Genechem) was injected at a rate of 50 nl/min. After injection, the syringe was left in place for 15 min and then withdrawn slowly. Subsequently, an electrode (Bio-Tech) was implanted on the skull and secured with dental cement. After full recovery and sufficient viral expression, EEG, EMG, and locomotor activity signals were recorded using a tremor detector (Medusa, Bio-Signal).

### Statistical Analysis

Tissue level of serotonin system, tremor detection of mice, and electrophysiological parameters were evaluated using GraphPad Prism9 (GraphPad Software). A two-tailed Student’s *t* test was used for the comparison of two groups, while one-way analysis of variance (ANOVA) was used for comparisons involving more than two groups. The homogeneity of variance was assessed using Brown-Forsythe and Bartlett’s tests. All data were presented as mean ± SEM (standard error of the mean), with ‘n’ representing the sample number (*i.e*., the number of independent experiments or cell numbers). Significant differences were denoted as ∗*p* < 0.05,∗∗*p* < 0.01,∗∗∗*p* < 0.001, and ∗∗∗∗*p* < 0.0001.

## Results

### Rhythmic Activity Detection of Mice Induced by Harmaline

**Fig. 1:**
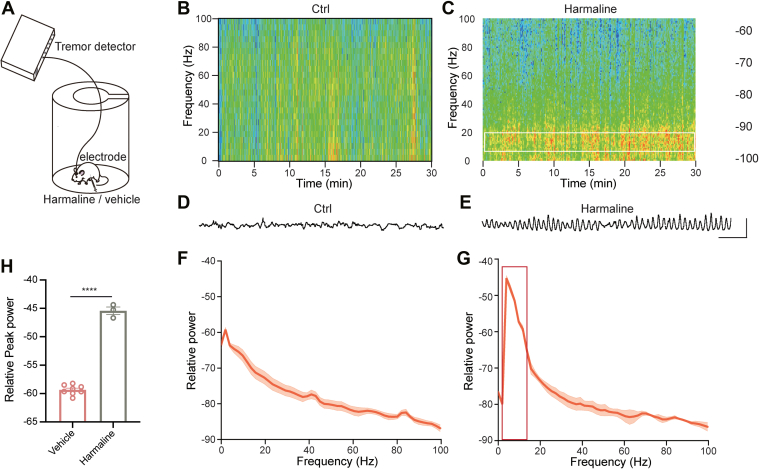
**Establishment and phenotypic characterization of the harmaline-induced mouse tremor model.***A*, Schematic paradigm for detection of harmaline-induced essential tremor. *B–C*, Representative spectrograms calculated from 30-min tremor measurements in mice based on head vibration respectively in control group (*B*) and harmaline-exposure group (*C*). *D–E*, Representative raw oscillation traces from head vibration measurement in control (*D*) and harmaline-exposure (*E*) mice. *F–G*, Average power spectrum from data in (*B*) and (*C*). n = 8 for control group (*F*) and n = 3 for harmaline-exposure group. *H*, comparison of the average peak power from 8 to 20 Hz between control (n = 8) and harmaline-exposure (n = 3) group. Data are presented as mean ± SEM. Ctrl, −59.34 ± 0.3005, 95% CI [−59.71, −59.29]; Harmaline, −45.43 ± 0.6715, 95% CI [−48.32, −42.54]. ∗∗∗∗*p* < 0.0001, two-tailed Students’ *t* test.

We established a harmaline-induced tremor model in mice following the described protocols from previous studies (6, 16, 17) (Fig. 1*A*). Upon injection of harmaline (dissolved in DMSO and diluted with saline, 30 mg/kg), mice exhibited pronounced tremors manifesting across the head, trunk, and limbs, compared with control littermates injected with the vehicle (DMSO diluted with saline). The tremors presented in harmaline-injected mice occurred within a distinct frequency range of 8 to 20 Hz, initiating approximately 3 min after intraperitoneal injection (Fig. 1, *B*–*G*). Additionally, harmaline-treated mice displayed significantly higher power in the 8 to 20 Hz frequency band in contrast with the control group (Fig. 1*H*). The tremors persisted for approximately 2 h, reaching their peak at 30 min post-injection. Subsequently, we sacrificed the model mice 30 min after harmaline administration and dissected the cerebellar cortex for subsequent protein and RNA extraction.

### Transcriptomic and Proteomic Workflow and Overall Characterization following Harmaline-Induced ET Model

**Fig. 2:**
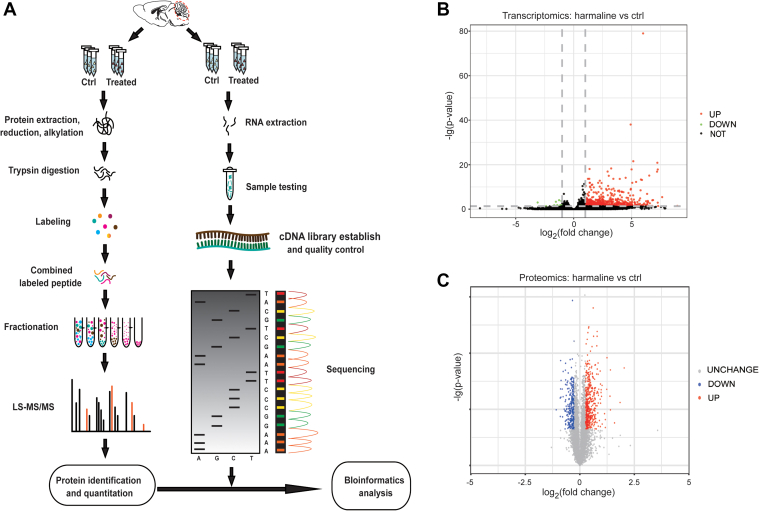
**Transcriptomic and proteomic workflow and preliminary screening of candidate genes involved in harmaline-ET.***A*, schematic workflow of RNA-seq and LC-MS/MS and joint bioinformatic analysis based on mouse cerebellar cortex. After vehicle or harmaline treatment, mouse cerebellar cortexes were dissected, followed by independent mass spectrometry and bulk RNA-Seq and combined analysis. *B*, 35125 genes were identified in transcriptomic analysis, including 614 up-regulated genes and 18 down-regulated genes. *C*, 5661 proteins were identified from proteomic analysis, including 469 upregulated proteins and 271 down-regulated proteins.

**Supplementary Figure 1:**
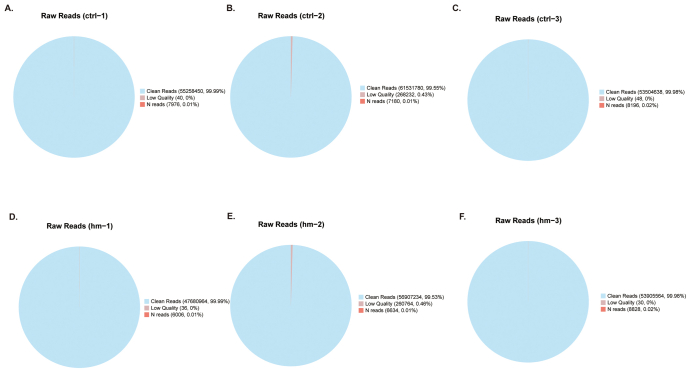
**Pie chart of Raw and clean reads of 6 samples.** (*A*) Raw and clean reads of the cerebellar cortex of control mouse-1, (*B*) Raw and clean reads of the cerebellar cortex of control mouse-2, (*C*) Raw and clean reads of the cerebellar cortex of control mouse-3, (*D*) Raw and clean reads of the cerebellar cortex of harmaline-injected mouse-1, (*E*) Raw and clean reads of the cerebellar cortex of harmaline-injected mouse-2, (*F*) Raw and clean reads of the cerebellar cortex of harmaline-injected mouse-3.

To date, no study has reported transcriptomic or proteomic changes in the cerebellar cortex of harmaline-administrated mice for the ET model. As illustrated in the schematic workflow (Fig. 2*A*), we conducted both transcriptomic and proteomic analysis, respectively, comparing 3 mice treated with harmaline with 3 vehicle-treated littermates so as to unravel the changes in transcriptomic and proteomic profiles associated with harmaline administration. For each cerebellar cortex lysate sample, we performed LC-MS/MS analysis in triplicate. The reliability of our data is supported by the high technical reproducibility and the minimal variation across cerebellar cortex samples. For transcriptomic analysis, all RNA samples met stringent criteria for cDNA library construction: OD_260/280_ = 2.1, RINs (RNA Integrity Number) ranged from 8.9 to 9.2, all Q-phred of 6 samples reached Q40), the least raw read of 6 samples was 47687006 and the least clean reads of 6 samples was 47680964 (Supplemental Fig. S1, *A*–*F*).

In total, we identified 35,125 genes from RNA-seq and 5661 proteins from LC-MS/MS. Through gene and protein difference analysis (Fig. 2, *B* and *C*), it is noteworthy that in the transcriptomic analysis, 614 genes were up-regulated, 18 genes were down-regulated; in comparison, 469 proteins were up-regulated, 271 proteins were down-regulated for proteomic analysis. However, the correlation of the gene expression level between these up-regulated and down-regulated genes and proteins in harmaline-treated mice remains unclear at this point.

### Co-occurring Alterations in the Cerebellum of Harmaline-Induced ET Identified Through Integrated Analysis of Transcriptomics and Proteomics

**Fig. 3:**
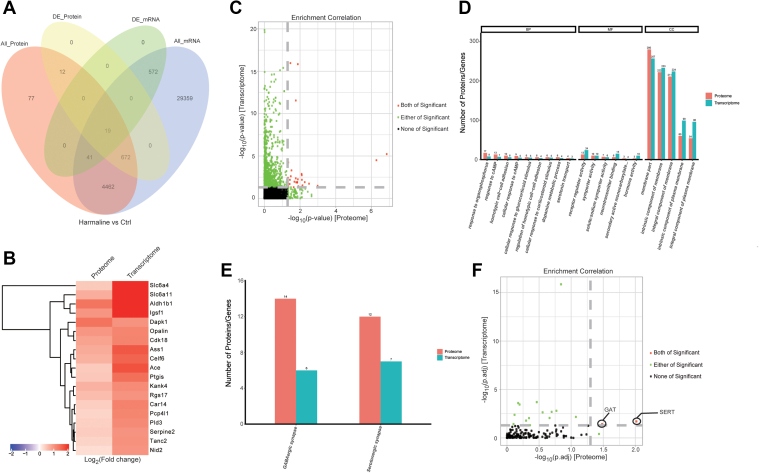
**Integrated informatic analysis based on transcriptomic and proteomic data.***A*, Integrated analysis revealed 5194 correlated genes with 19 significant upregulation. *B*, the profile and expression richness of 19 genes based on clustering analysis. *C*, GO analysis of enrichment correlation between proteome and transcriptome. *D*, combined GO analysis of enrichment correlation between proteome and transcriptome. *E*, combined KEGG analysis of enrichment correlation between proteome and transcriptome. *F*, KEGG analysis of enrichment correlation between proteome and transcriptome.

**Supplementary Figure 2:**
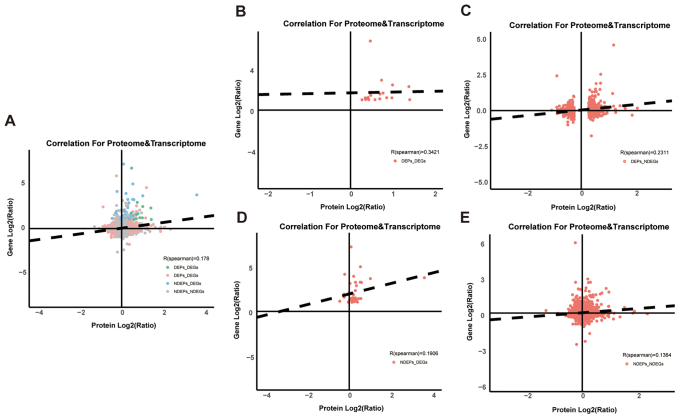
**Correlation analysis of Proteome and Transcriptome result.** (*A*) Combined correlation analysis of Proteome and Transcriptome result of all data, (*B*) Correlation analysis of Proteome and Transcriptome result of differentially expressed proteins and genes (DEP and DEG), (*C*) Correlation analysis of Proteome and Transcriptome result of differentially expressed proteins and non-differentially expressed genes (DEP and NDEG), (*D*) Correlation analysis of Proteome and Transcriptome result of non-differentially expressed proteins and differentially expressed genes (NDEP and DEG), (*E*) Correlation analysis of Proteome and Transcriptome result of non-differentially expressed proteins and non-differentially expressed genes (NDEP and NDEG).

We then conducted the correlation analysis between Proteome and Transcriptome results (supplemental Fig. S2), a weak but positive correlation was identified between those two datasets. Further, we integrated transcriptomic and proteomic information derived from the same treatment, considering genes that exhibited co-directional changes in transcription and translation as the correlated ones. In this context, 5194 correlated genes were identified, among which 19 were deemed significant, all showing upregulation (Fig. 3*A*). Subsequently, a clustering analysis of these significantly correlated genes revealed a consistent pattern of upregulation across all 19 (Fig. 3*B*).

To gain insights into the functional implications of those correlated genes, we conducted enrichment analysis. GO enrichment analysis identified over 30 significant GO terms that exhibiting concordance in both omics data (Fig. 3*C*). Notably, among these terms, proteins and genes located at the cell membrane are considered to undergo most significant changes (Fig. 3*D*). In KEGG enrichment, only the serotonin synapse and GABA synapse pathways stood out as highly significant in both transcriptomic and proteomic analyses (Fig. 3*E*). Specifically, two genes: serotonin transporter (SERT) and GABA transporter (GAT) were identified as the most significant ones (Fig. 3*F*). This suggests that SERT and GAT may play a significant role in harmaline-induced tremor.

### The Pivotal Role of Serotonin Transporter of Cerebellar Cortex in Harmaline-Induced Tremor

**Fig. 4:**
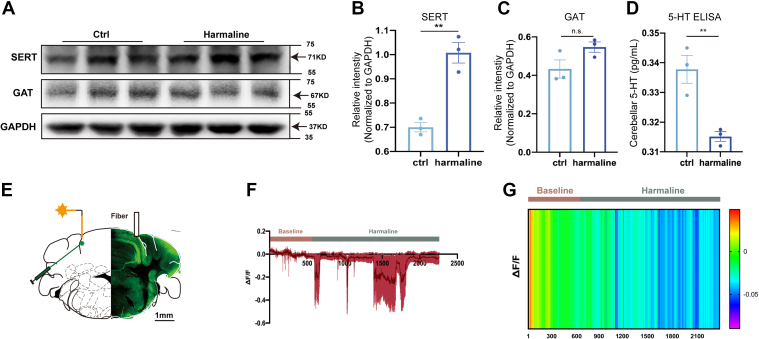
**Regulation of SERT expression and 5-HT level in harmaline-induced tremor.***A–C*, Representative blots (*A*) and quantification of SERT (*B*) and GAT (*C*) from cerebellar cortex in mice following vehicle or harmaline treatment as indicated. GAPDH was used as a cytoplasmic protein control. The protein level was presented as mean ± SEM. For GAT, *p* = 0.1039, no significance between two groups (ctrl, 0.4331 ± 0.0473, 95% CI [0.239, 0.637]; harmaline, 0.5471 ± 0.268, 95% CI [−0.61, 1.70]). For SERT, ∗∗*p* < 0.01 (ctrl, 0.7003 ± 0.0192, 95% CI [0.618, 0.783]; harmaline, 1.008 ± 0.426, 95% CI [−0.82, 2.84]). Three independent experiments. Two-tailed Students’ *t* test. *D*, ELISA quantification of 5-HT content in mouse cerebellar cortex 30 min after vehicle/harmaline treatment. Data are presented as mean ± SEM. Based on the standard curve, the 5-HT concentration: ctrl, 0.3378 ± 0.0047, 95% CI [0.318, 0.358]; harmaline, 0.3152 ± 0.0016, 95% CI [0.3083, 0.3221]. ∗∗*p* < 0.01. Three independent experiments. Two-tailed Students’ *t* test. *E*, schematic diagram for 5-HT sensor transfection and fiber photometry. *F*, Quantification analysis of 5-HT sensor fluorescent fluctuation following harmaline treatment. n = 4 mice. *G*, representative heatmap of time-lapse recording of 5-HT sensor fluorescence.

To verify the potential function of SERT and GAT in harmaline-induced tremor, we conducted Western Blot analysis. Using the stable mouse model of harmaline-induced ET, we observed an increase in SERT protein levels in the mouse cerebellar cortex compared to the vehicle control group, which aligns with our integrated analysis of transcriptomics and proteomics (Fig. 4, *A* and *B*). However, despite an upward trend, the quantification of GABA transporter (GAT) showed no significant difference between the two groups (Fig. 4, *A*–*C*), indicating that SERT may play a more crucial role in harmaline-induced tremor.

To further investigate the relationship between SERT and serotonin (5-HT) in harmaline-induced tremor, we applied Enzyme-Linked Immunosorbent Assay (ELISA) and found a significant down-regulation of 5-HT in the cerebellum during harmaline-induced tremor (Fig. 4*D*). We then locally expressed genetic sensor of 5-HT in the cerebellar cortex by AAV (AAV-hSyn-5HT3.0) to continuously monitor the fluctuation of 5-HT with high temporal and spatial resolution (Fig. 4*E*). The results revealed that within 30 min of harmaline administration (intraperitoneal), the content of 5-HT declined with fluctuations (Fig. 4, *E* and *G*), confirming the ELISA findings. This suggests that the down-regulation of 5-HT may be one of the consequences of the increase in SERT.

### Targeted Inhibition of SERT Reverses the Excitability and Tremor Induced by Harmaline

**Fig. 5:**
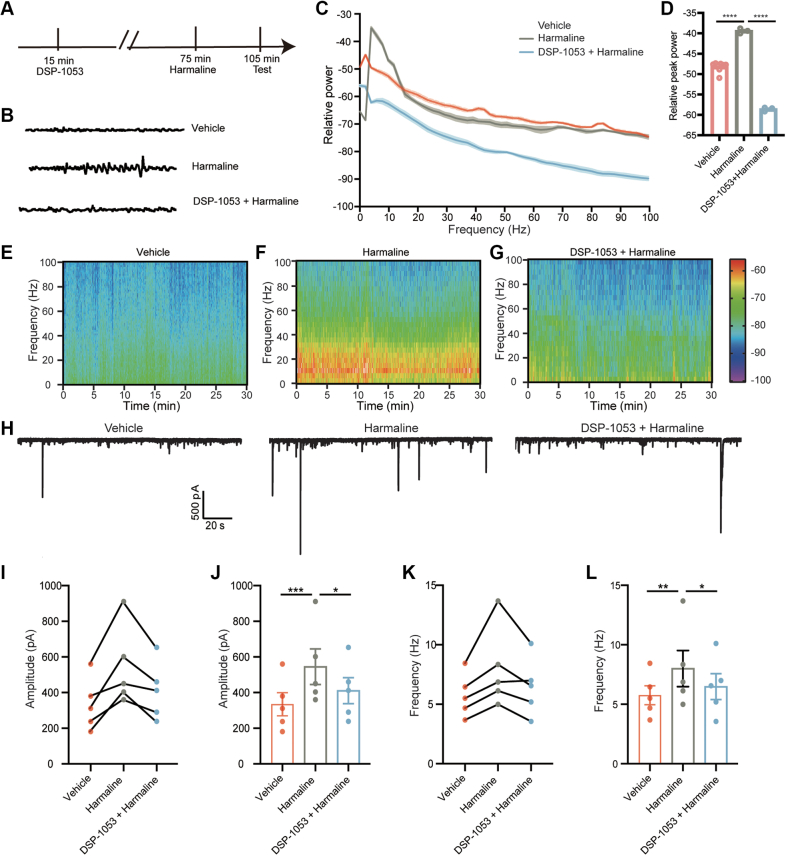
**The cellular and molecular mechanisms underlying harmaline-induced tremor.***A*, schematic paradigm for behavioral experiments targeting SERT inhibition. *B*, representative raw data of head vibration using tremor detector in vehicle, harmaline with or without DSP-1053 treatment group. *C–D*, average power spectrum from data in (*B*). n = 8 mice for control group, n = 3 mice for harmaline-exposure group and n = 3 mice for harmaline with DSP-1053 group. *D*, comparison of average peak power from 8 to 20 Hz among control (n = 8), harmaline (n = 3) and harmaline with DSP-1053 group (n = 3). Data are presented as mean ± SEM, Ctrl, −48 ± 0.4252, 95% CI [−49.01, −46.99]; Harmaline, −39.82 ± 0.6153, 95% CI [−42.47, 37.17]; Harmaline + DSP-1053, −58.6 ± 0.2877, 95% CI [−59.84, −57.36]. ∗∗∗∗*p* < 0.0001, one-way ANOVA. *E–G*, representative spectrograms calculated from 30-min tremor measurements based on head vibration, respectively, in control group, harmaline group and harmaline + DSP1053 group. *H*, Representative traces of spontaneous excitatory postsynaptic current (sEPSC) on cultured primary cerebellar neurons at DIV10. *I–J*, Amplitude quantification of sEPSC, data are presented as mean ± SEM. Ctrl, 334.4 ± 65.66, 95% CI [152.1, 516.7]; Harmaline, 545.6 ± 100.1, 95% CI [267.6, 823.5]; Harmaline + DSP-1053, 410.9 ± 72.74, 95%CI [208.9, 612.8]. *K–L*, frequency quantification of sEPSC, data presented as mean ± SEM, Ctrl, 5.752 ± 0.8128, 95%CI [3.495, 8.009]; Harmaline, 8.002 ± 1.520, 95%CI [3.782, 12.22]; Harmaline + DSP-1053, 6.480 ± 1.087, 95%CI [3.462, 9.498]. n = 5 cells. ∗*p* < 0.05, ∗∗*p* < 0.01, ∗∗∗*p* < 0.001, one-way ANOVA.

To functionally elucidate the role of SERT in harmaline-induced tremor, we conducted behavioral experiments in mice by inhibiting SERT. The mice were pre-injected with DSP-1053, an inhibitor of SERT, 1 h before harmaline administration (Fig. 5*A*). Remarkably, with DSP-1053 pre-treatment, the harmaline-induced tremors were significantly attenuated in mice (Fig. 5, *B*–*D*). These results emphasize the critical role of SERT in harmaline-induced tremor. To further investigate the potential mechanism of harmaline-induced tremor and targeted inhibition of SERT, we conducted electrophysiology experiments on cultured primary cerebellar cortical neurons. Using the whole-cell patch clamp technique, we observed that harmaline perfusion enhanced the activity of Purkinje cells (PCs) by increasing spike firing, both in amplitude and frequency (Fig. 5, *H*–*L*), confirming the impact of harmaline on PC activity. Importantly, pre-perfusion with DSP-1053 significantly reversed the effect of harmaline, highlighting the involvement of SERT in harmaline-enhanced cerebellar activity (Fig. 5, *H*–*L*). These results demonstrated that SERT plays a pivotal role in harmaline-induced tremor by affecting PC excitability.

**Fig. 6:**
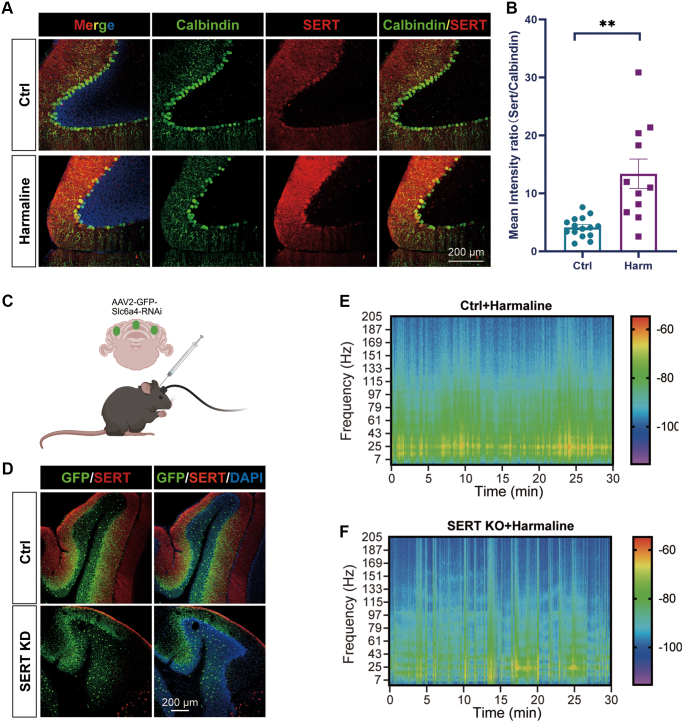
**The significant role of SERT in Purkinje cells.***A*, representative immunofluorescence image of the expression change of SERT in cerebellar Purkinje cells following harmaline treatment. The scale bar, 200 μm. *B*, quantification of (*A*). Ctrl, n = 15 slices, Harmaline, n = 10 slices. Data are presented as mean ± SEM. Ctrl, 4.143 ± 0.4531, 95% CI [3.172, 5.115]; Harmaline, 13.38 ± 2.536, 95% CI [7.727, 19.03]. ∗∗*p* < 0.01. Two-tailed Students’ *t* test. *C*, Schematic illustration of cerebellar SERT knockdown and electrode implant for tremor detection. *D*, representative images of AAV-delivered miRNA-mediated SERT knockdown. *Green* fluorescence for AAV-infected neurons via hSyn1 promoter, *red* fluorescence for SERT signal. The scale bar, 200 μm. *E–F*, representative spectrograms calculated from 30-min tremor measurements based on head vibration respectively in control group and SERT knockout group following harmaline exposure. Ctrl + harmaline, n = 7 mice; SERT-KO + Harmaline, n = 9 mice. Abbr: Ctrl: control, Harm: Harmaline, KO: knockout; SERT, serotonin transporter.

To examine the spatial expression and distribution of SERT in the cerebellum, immunohistochemical staining was performed on mouse cerebellum sections. We observed abundant expression of SERT within the cerebellum cortex, with pronounced enrichment in the dendritic arbors and somata of Purkinje cells (Fig. 6*A*). Notably, harmaline administration resulted in a significant upregulation of SERT expression in the cerebellum (Fig. 6*B*). While the above pharmacological findings support a role for SERT in harmaline-induced tremor, we further employed a genetic strategy to validate these results. An AAV vector encoding miRNA for SERT knockdown was constructed and delivered into the cerebellar cortex (Fig. 6*C*). Infection with this virus significantly reduced SERT levels in GFP-positive neurons (Fig. 6*D*). Importantly, miRNA-mediated knockdown of SERT across multiple cerebellar regions markedly attenuated harmaline-induced tremor (Fig. 6, *E* and *F*). Together, these results reinforce the novelty of our study, which identifies SERT as a potential therapeutic target for essential tremor.

## Discussion

Mounting evidence implicates alterations in the cerebellum in ET. Postmortem examinations of ET patients have revealed characteristic neuropathological changes, including torpedo-shaped swelling of neuronal branches (18) and decreased density in Purkinje cells (19), as well as changes in hairy baskets for basket cells (20), among others. Drugs (including propranolol, primidone, etc.) are the main therapy for ET, while drugs can be only effective for 1/3 patients. Thus, finding novel targeted therapies can be an urgent for ET (21). Other potential treatments are under exploration currently, such as memantine (NMDA receptor antagonist) (22), tiagabine (GAT-1 inhibitor) (23), while those treatments have not been fully established and the safety issues are warrant to be determined.

In laboratory animals, harmaline-induced tremor serves as a recognized model for providing a valuable tool for qualitatively assessing the effectiveness of pharmaceutical interventions for ET. As reported, harmaline-induced tremor can be distinguished from ET mainly as it is an acute model while ET is a chronic and possibly neurodegenerative disorder (7), while in this study, we mainly focus on searching the novel targeted therapeutic methods for ET by revealing its underlying mechanism.

Our approach, which combines proteomic and transcriptomic sequencing technologies, introduces a novel dimension to elucidating gene signatures by reducing the false discovery rates inherent in individual technologies and utilizing a more precise bioinformatics infrastructure (24, 25). Within our dataset, the transcriptomic sequencing results were used as contrast version for proteomic analysis results to find the overlay results, the high-specificity turned out significant up-regulation of 19 genes along with their associated proteins. Through KEGG pathway analysis, we identified two specific genes, GAT and SERT. However, our Western blot results revealed a disparity between the two. Unlike SERT, the quantity of GAT did not show a significant increase after harmaline treatment. This discrepancy may be attributed to the lower precision of WB, as it only reflects proteins with marked variations at the protein level.

SERT, a component of the serotonin system, is known for its involvement in various neuropsychiatric disorders, including depression, bipolar disorder, anxiety, and neurodegenerative conditions (26). The structural similarity between β-carboline alkaloid and serotonin has been reported (27). However, the role of the serotonin system in harmaline-induced tremor, particularly in olivocerebellar function, remains a highly debated topic (28, 29, 30, 31), with limited research highlighting its role in ET. Our research group made a pioneering discovery by revealing a conspicuous up-regulation of SERT in harmaline-induced tremor, suggesting its potential as a key regulatory factor in this context.

To strengthen the validity of our findings, we employed multiple experimental methods including WB, ELISA, and optical fiber recording. The collective results consistently confirmed that harmaline induces the upregulation of SERT, leading to an increase in serotonin uptake and subsequently causing a decrease in cerebellar serotonin levels. Furthermore, we conducted electrophysiological assessments on cultured primary cerebellar cortical neurons and observed tremors in mice. These experiments demonstrated that the excitability of neurons and harmaline-induced tremors can be significantly suppressed. This further validated the critical role of SERT in harmaline-induced tremor. To provide additional support for this conclusion, we utilized DSP-1053, an inhibitor of SERT, and observed its effect on tremor suppression. The application of DSP-1053 confirmed the involvement of SERT in harmaline-induced tremor. And with the further application of SERT knockdown *in vivo*, the attenuated tremor after harmaline-injected strength our theory about the pivotal role of SERT in harmaline-induced tremor and the higher expression of SERT in Purkinje cells further revealed its specific role in Purkinje cells.

Our findings align closely with previous studies that have suggested the potential effectiveness of serotonergic agonists, such as trazodone, in ameliorating ET (32, 33, 34, 35). While significant improvements were observed in only two small clinical trials (involving 2 patients (33) in one study and 5 out of 6 patients (34) in another), our results make a contribution to the increasing body of evidence supporting the involvement of the serotonin system in ET. These collective findings emphasize the potential relevance of targeting the serotonin system as a therapeutic approach for ET.

Selective serotonin reuptake inhibitors (SSRI) are one of the most frequent choices for depression and anxiety-related disorders for its rather safe characteristics while with caution use in pregnant woman; literature indicates that the use of SSRIs may lead to several neonatal complications (36). Nonetheless, some published articles have presented contrasting views. For instance, citalopram, an SSRI, has been reported to augment harmaline-induced tremor (37). Additionally, after harmaline injection, elevated level of 5-HT was observed in the striatum, cortex, hypothalamus, hippocampus (38, 39) and brainstem (37). Furthermore, sertraline, escitalopram, another SSRI, was implicated in inducing movement disorders, including dystonia, akathisia, and Parkinsonian symptoms in a 38-year-old male patient (40).

Several factors may contribute to the discrepancies noted above. Firstly, previous studies did not specifically focus on serotonin level in the cerebellar cortex, where our findings revealed a decline in 5-HT. Moreover, the observed augmentation in climbing fiber reuptake of 5-HT matches with the elevation of 5-HT in the brainstem. Additionally, citalopram exhibits low affinity for various receptors (37), including SERT, dopamine receptors and monoamine oxidase inhibitor receptors. As the citalopram dosage rise, the inhibition of these receptors could contribute to the augmentation of tremors. Conversely, it is theorized that SSRI may lead to a further reduction of serotonergic activity in brainstem, enhancing glucose metabolism and adenosine, eventually bringing about tremors (37).

Lastly, our study has collected evidence to support the critical role of SERT in harmaline-induced tremor, nevertheless, it is important to note several limitations of our study, including the small sample size used for the transcriptomic and proteomic analyses, the uneven sample size in some behavioral experiments, and the use of a single ET mouse model. Future work should involve larger and balanced sample sizes for behavior test following SERT manipulation in multiple ET animal models, and investigations into the translational potential of SERT targeting for ET treatment.

## Conclusion and Perspective

The current study employed transcriptomic and proteomic analysis to elucidate alterations within cerebellar cortex following harmaline treatment in mice. Our findings confirm the up-regulation of SERT, a component of the serotonin system, in harmaline-induced tremor. This highlights a potential role for SERT in the pathogenesis of ET, offering a novel research strategy and identifying a potential molecular target for further investigation. Moving forward, several avenues for further research are suggested. Firstly, it is crucial to demonstrate the molecular pathway of SERT in the pathophysiology of ET. Secondly, exploring the potential of 5-HT in peripheral blood, and additionally confirm it in human autopsy samples as an auxiliary indicator for the diagnosis of ET could enhance diagnostic approach. In addition, considering the widespread use of 5-HT reuptake inhibitors in depression, a similar therapeutic targeting SERT could be investigated for its applicability in ET treatment within its safety limitation.

## Data Availability

URL: https://www.ebi.ac.uk/pride/login

Project ID: PXD055985.

Username: consentir@126.com,

Password: abc123456

## Supplemental data

This article contains supplemental data

## Conflict of Interest

The authors declare that they do not have any conflicts of interest with the content of this article.
